# *Porphyromonas somerae* Invasion of Endometrial Cancer Cells

**DOI:** 10.3389/fmicb.2021.674835

**Published:** 2021-07-23

**Authors:** Taylor A. Crooks, Joseph D. Madison, Dana M. Walsh, William G. Herbert, Patricio R. Jeraldo, Nicholas Chia, William A. Cliby, Scott H. Kaufmann, Marina R. S. Walther-Antonio

**Affiliations:** ^1^Microbiome Program, Center for Individualized Medicine, Mayo Clinic, Rochester, MN, United States; ^2^Department of Microbiology and Immunology, University of Minnesota Twin Cities, Minneapolis, MN, United States; ^3^Biology Department, University of Massachusetts Boston, Boston, MA, United States; ^4^Department of Surgery, Mayo Clinic, Rochester, MN, United States; ^5^Mayo Clinic Graduate School of Biomedical Sciences, Rochester, MN, United States; ^6^Department of Obstetrics and Gynecology, Mayo Clinic, Rochester, MN, United States; ^7^Division of Oncology Research, Department of Oncology, Mayo Clinic, Rochester, MN, United States

**Keywords:** *Porphyromonas somerae*, endometrial cancer, estradiol, intracellular invasion, succinate dehydrogenase, fumarate reductase, metabolomics

## Abstract

Recent evidence suggests an association between endometrial cancer and the understudied bacterial species *Porphyromonas somerae*. This association was demonstrated in previous work that indicated a significantly enriched abundance of *P. somerae* in the uterine microbiome of endometrial cancer patients. Given the known associations of the *Porphyromonas* genus and oral cancer, we hypothesized that *P. somerae* may play a similar pathogenic role in endometrial cancer via intracellular activity. Before testing our hypothesis, we first characterized *P. somerae* biology, as current background data is limited. These novel characterizations include growth curves in liquid medium and susceptibility tests to antibiotics. We tested our hypothesis by examining growth changes in response to 17β-estradiol, a known risk factor for endometrial cancer, followed by metabolomic profiling in the presence and absence of 17β-estradiol. We found that *P. somerae* exhibits increased growth in the presence of 17β-estradiol of various concentrations. However, we did not find significant changes in metabolite levels in response to 17β-estradiol. To study direct host-microbe interactions, we used *in vitro* invasion assays under hypoxic conditions and found evidence for intracellular invasion of *P. somerae* in endometrial adenocarcinoma cells. We also examined these interactions in the presence of 17β-estradiol but did not observe changes in invasion frequency. Invasion was shown using three lines of evidence including visualization via differential staining and brightfield microscopy, increased frequency of bacterial recovery after co-culturing, and *in silico* methods to detail relevant genomic and transcriptomic components. These results underscore potential intracellular phenotypes of *P. somerae* within the uterine microbiome. Furthermore, these results raise new questions pertaining to the role of *P. somerae* in the progression of endometrial cancer.

## Introduction

The prevalence of endometrial cancer (EC) has increased steadily over the past two decades. From 1999 to 2017, new cases of EC have risen from 35,000 to 57,000 in the United States (Group, 2019). However, the change in mortality rate from 1999 to 2017 is small, only decreasing from 5.4 to 5.21% (Group, 2019). Globally, EC is the sixth most frequently diagnosed cancer and 14th most fatal (Bray et al., 2018). The major risk factors identified in the development of EC include unopposed estrogen therapy, metabolic syndrome (obesity and type II diabetes) and genetic predisposition, including Lynch and Cowden syndromes (Strom et al., 2006; Njoku et al., 2020). While no direct mechanistic causes of EC have been determined, recent microbiome findings may contribute to the understanding of endometrial cancer etiology.

Previous studies conducted by our group have demonstrated an overrepresented abundance of the bacterial species *Porphyromonas somerae* in patients with EC relative to patients with benign uterine disease via 16S rRNA gene sequencing (Walther-António et al., 2016; Walsh et al., 2019). While limited work has been published on *P. somerae*, the well described taxonomic relative, *Porphyromonas gingivalis* has been associated with cancer (Lafuente Ibáñez de Mendoza et al., 2020). Known for its association to periodontal disease, *P. gingivalis* has also been shown to evade host-immune cells via intracellular invasion and induce anti-apoptotic effects in human cells (Lamont et al., 1995; Yilmaz et al., 2008). The taxonomic and functional relatedness of *P. gingivalis* and *P. somerae* therefore provides an additional impetus for examining the role of *P. somerae* in EC progression.

In order to consider the plausibility of the shared virulence factors between *P. somerae* and *P. gingivalis*, it is helpful to understand the mechanisms through which *P. gingivalis* invades human cells. *P. gingivalis* uses a gingipain system in order to adhere to epithelial cells in the oral cavity (Chen et al., 2001). Upon contact with host epithelial cells, *P. gingivalis* produces the internalin, InlJ, which is essential for monospecies biofilm formation (Capestany et al., 2006). Additionally, contact with epithelial cells can potentially drive degradation of epithelial cell junctional complexes such as E-cadherin by *P. gingivalis* (Katz et al., 2000; Capestany et al., 2006). After traversing the extracellular matrix, intracellular invasion can occur. Once inside the epithelial cells, endopeptidases are essential for *P. gingivalis* survival and are controlled by *pepO* gene expression (Park et al., 2004). The production of ATP via the electron transport chain in *P. gingivalis* is posited to be fumarate dependent using fumarate reductase and succinate dehydrogenase (Meuric et al., 2010). Succinate dehydrogenase is upregulated following epithelial cell invasion resulting in prolyl hydroxylase domain inhibition (Su et al., 2020). While these elements have been described in *P. gingivalis* they have yet to be outlined as important components for virulence in the less characterized *Porphyromonas* species, *P. somerae*.

Now known to be prevalent in a subset of EC patients, the pigmented gram-negative anaerobic rod species *P. somerae* has been historically found in chronic soft tissue or bone infections (Summanen et al., 2005). In an original study detailing 58 isolates of *P. somerae*, all strains were susceptible to cefoxitin, imipenem, and metronidazole (Summanen et al., 2005). Most strains were susceptible to clindamycin, and a smaller proportion were susceptible to amoxicillin. 21% of strains were found to produce β-lactamase, indicative of β-lactam resistance. Gentamicin susceptibility in *P. somerae* has not been reported; however, gentamicin resistance has been reported in *P. gingivalis* (Malek et al., 1994). While some resistance has been observed in *P. somerae*, we recently characterized the transcriptome via bulk and single-cell RNA-seq finding various transcripts conferring virulence *in vitro* (Liu et al., 2019). The drivers of these observed changes in expression nonetheless remain largely uncharacterized. Such factors may be driven by host and host-microbiome components such as nutrient availability or hormonal status. One example of such hormonal effects has been shown in *Pseudomonas aeruginosa* isolates from cystic fibrosis patients, where *P. aeruginosa* was found to be sensitive to increased estrogens resulting in increased virulence phenotypes such as mucoid conversion (Chotirmall et al., 2012). Relatedly, there has been evidence that estrogen can alter metabolic states such as succinate production in *Porphyromonas* (Kornman and Loesche, 1982). Since bacterial virulence has been found to be exacerbated by shifts in sex hormones in other organisms (García-Gómez et al., 2013), fluctuating hormones such as estradiol may promote a virulence role of *P. somerae* within the uterine microbiome.

Estradiol levels fluctuate across the hormonal cycle in healthy individuals who undergo regular menstrual cycles. The follicular phase is characterized by rising estradiol levels and peaks during the ovulatory phase. When pregnancy does not occur following the luteal phase, estradiol concentrations decrease to basal levels followed by the shedding of the endometrium (Knudtson and McLaughlin, 2016). During pregnancy, estradiol levels steadily increase, peaking in the third trimester and returning to pre-pregnancy levels within 5 days of parturition (Malek et al., 1994; Schock et al., 2016). In perimenopausal individuals, ovarian function declines leading to a decrease in systemic estradiol levels comparable to that of age-matched XY individuals (Yasui et al., 2012). As a result of these various hormonal shifts, concentrations of serum estradiol can range from 20,000 pg/mL to <20 pg/mL (Stanczyk and Clarke, 2014). Uterine endometrium and breast cancer tissue can sequester as much as 10–50 times the amount of estradiol relative to serum levels in postmenopausal women (Recchione et al., 1995; Pasqualini et al., 1996; Zhu and Conney, 1998; Gruber et al., 2002). Unopposed estrogen therapy has been identified as a risk factor for the development of endometrial cancer (Grady et al., 1995) and therefore replaced in clinical practice by combined estrogen-progestin therapy (Pike et al., 1997). As these hormonal states and concentrations vary, it is possible that increasing estradiol levels could promote *P. somerae* pathogenicity within the uterine microbiome.

We therefore hypothesized that *P. somerae* undergoes intracellular invasion like that of *P. gingivalis* and that this activity may be promoted by increasing 17β-estradiol. Here we characterize *P. somerae* growth and metabolism to better understand basic *P. somerae* biology. We then test the effects of 17β-estradiol on *P. somerae* interactions with the endometrial cells via growth experiments, *in vitro* invasion assays, and *in silico* analysis. We conclude with a discussion of our results and their implication for future work.

## Results

### *Porphyromonas somerae* Achieves Log Phase Growth in Reduced Chopped Meat Broth and Is Susceptible to Gentamicin

Considering *Porphyromonas somerae* growth had not been previously characterized in liquid media, we sought to find a growth method that could rapidly propagate cells for experimentation. As other *Porphyromonas* species are readily cultured in reduced chopped meat broth, we hypothesized that *P. somerae* would also grow in this medium. We found that *P. somerae* achieves log phase growth at 37°C after an 8-h lag phase in pre-reduced chopped meat broth in anaerobic conditions (Supplementary Figure 1A). We also sought to determine what concentration of gentamicin, if any, inhibits *P. somerae* growth. When grown in anaerobic conditions at 37°C in pre-reduced chopped meat broth, we found that the minimum inhibitory concentration (MIC) of gentamicin is 100 μg/mL (Supplementary Figure 1A). We additionally sought to use an antibiotic with bacteriostatic activity and a low MIC for subsequent experiments using antibiotics. For this reason, we employed cefoxitin with a previously reported MIC of <4 μg/mL. In order to determine the approximate number of colony forming units (CFUs)/mL in a given solution relative to the OD_600_ measurement, a growth curve was performed with samples taken at regular intervals. Samples were plated onto CDC blood agar plates to determine relative CFUs/mL after incubation. We found that at an OD_600_ measurement of 1.0 from cultures in log phase, *P. somerae* yields approximately 1.3 × 10^9^ CFUs/mL (Supplementary Figure 1B).

### 17β-Estradiol Promotes Growth of *Porphyromonas somerae*

**FIGURE 1:**
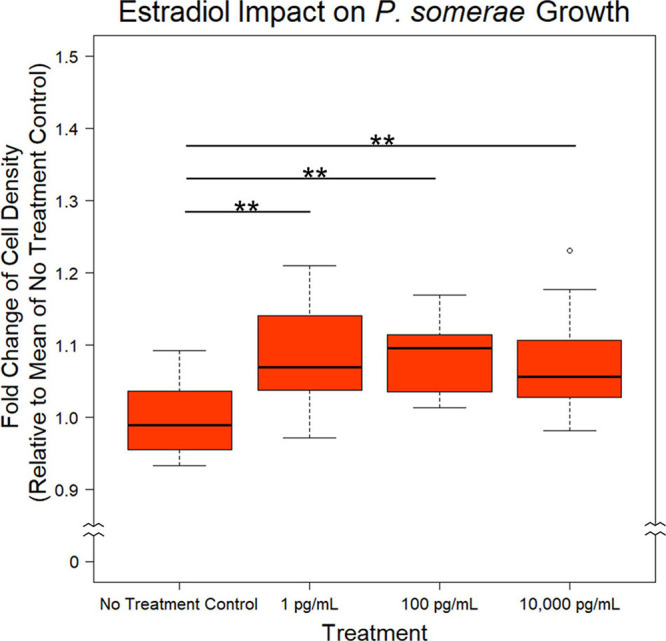
Fold change in *P. somerae* cell density after 8.5-h exposure to 17β-estradiol concentrations as indicated in pre-reduced chopped meat broth. Fold change determined by comparing OD_600_ measurement to the corresponding experimental mean OD_600_ of no 17β-estradiol treatment control. Experiment performed 3 times with *n* = 5 for each treatment for a total *n* = 15 for all samples. Outliers shown are values below or above 1.5× Interquartile Range from 1st or 3rd quartile respectively. Statistical significance calculated from Kruskal–Wallis test and Dunn’s test *post hoc* with α = 0.05. **P < 0.01.

After finding a suitable liquid medium for growth and experimentation, we investigated the impact of 17β-estradiol on *P. somerae* growth. While 17β-estradiol levels sequestered within tissues are difficult to assess, we recognize the potential for increased concentrations in endometrial tissue given its proximity to ovarian tissue. We therefore tested a wide range of 17β-estradiol concentrations including 1, 100, and 10,000 pg/mL. Relative to no added 17β-estradiol, *P. somerae* exhibits increased statistically significant growth in all concentrations of 17β-estradiol (Figure 1, *P* < 0.01).

### *Porphyromonas somerae* Produces Important Metabolic Intermediates and Short-Chain Fatty Acids

**FIGURE 2:**
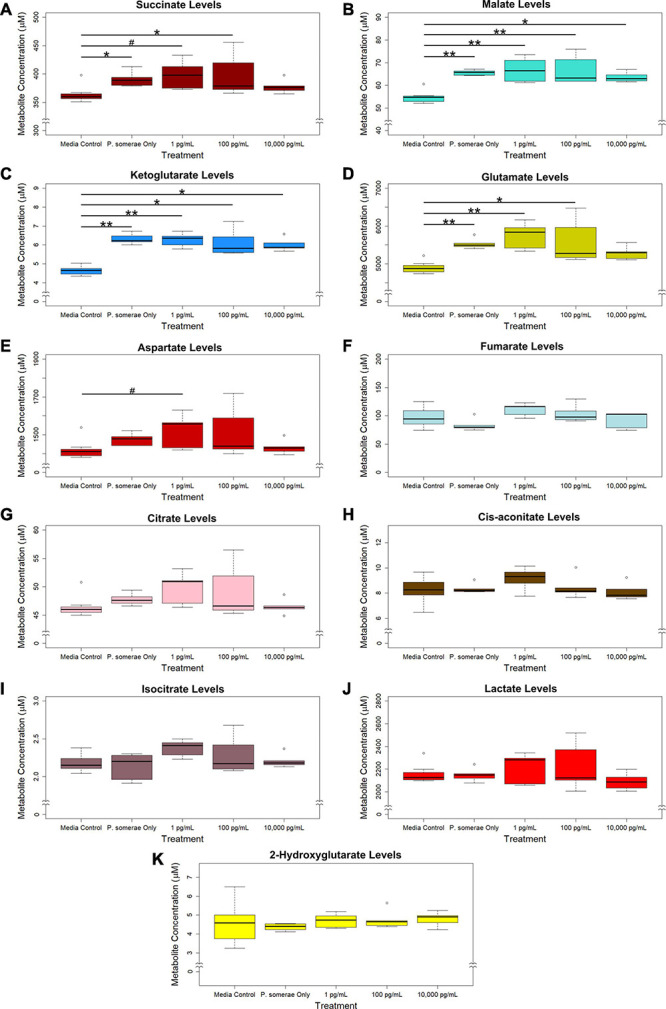
GC-MS analysis of citric acid cycle intermediates and lactate production from *Porphyromonas somerae* depleted media after 8.5-h exposure to 17β-estradiol concentrations of 1, 100, and 10,000 pg/mL as indicated. Outliers shown are values below or above 1.5 × Interquartile Range from 1st or 3rd quartile, respectively. Statistical significance calculated from Kruskal–Wallis test and Dunn’s test *post hoc* with α = 0.05. ^#^*P* = 0.1–0.05, *P < 0.05, **P < 0.01. Media control *n* = 8, *P. somerae* only *n* = 5, 1 pg/mL *n* = 5, 100 pg/mL *n* = 5, 10,000 pg/mL *n* = 5. **(A)** Succinate, **(B)** Malate, **(C)** Ketoglutarate, **(D)** Glutamate, **(E)** Aspartate, **(F)** Fumarate, **(G)** Citrate, **(H)** Cis-aconitate, **(I)** Isocitrate, **(J)** Lactate, **(K)** 2-Hydroxyglutarate.

Based on the increased growth in the presence of 17β-estradiol, we hypothesized that 17β-estradiol might induce changes in metabolic activity to promote increased growth. To assess this possibility, we compared metabolic profiles of *P. somerae* both in the presence and absence of 17β-estradiol at concentrations relevant to post-menopause, healthy individuals, and excess estrogen levels. After growing *P. somerae* in pre-reduced chopped meat broth, gas chromatography-mass spectrometry (GC-MS) was performed on depleted media to assess quantitative abundance of citric acid cycle intermediates present both in the presence and absence of 17β-estradiol. We found significantly increased levels of succinate, malate, ketoglutarate, and glutamate in the depleted media of *P. somerae* with no added 17β-estradiol relative to uninoculated media (Figures 2A–D, *P* < 0.05). Similarly, significantly increased levels of these intermediates were detected in samples with 17β-estradiol added at concentrations of 1 and 100 pg/mL relative to uninoculated media. Significantly increased metabolite production in 10,000 pg/mL of 17β-estradiol relative to uninoculated media was only observed for malate and ketoglutarate. Aspartate levels were found to be enriched in samples of 1 pg/mL of 17β-estradiol relative to uninoculated media; however, no significance was observed (Figure 2E, *P* > 0.05). No significant changes were observed in fumarate, citrate, cis-aconitate, isocitrate, lactate, or 2-hydroxyglutarate levels (Figures 2F–K, *P* > 0.05). No significant changes in citric acid cycle intermediates or lactate were observed between 17β-estradiol levels.

**FIGURE 3:**
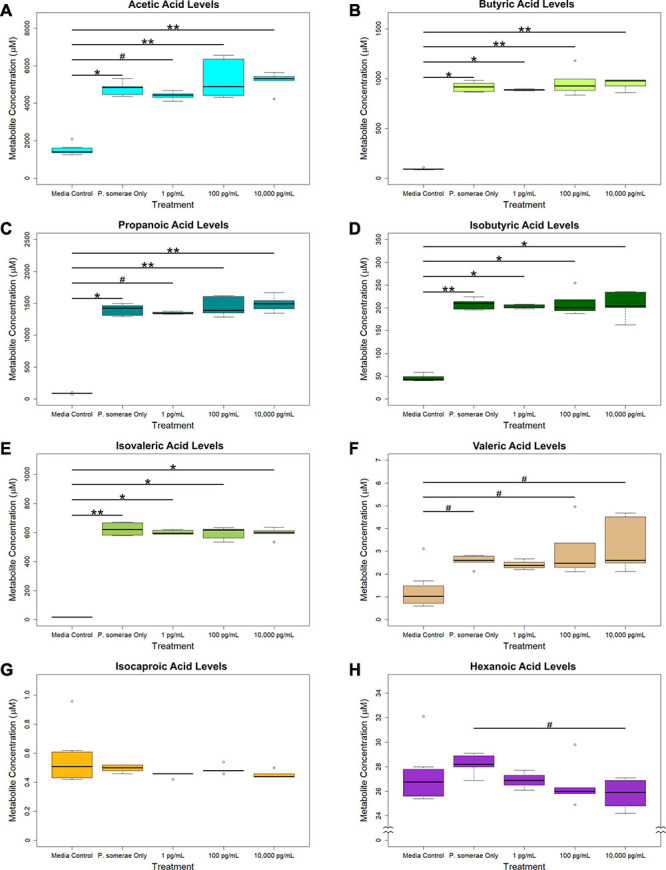
GC-MS analysis of short chain fatty acid production from *Porphyromonas somerae* depleted media after 8.5-h exposure to 17β-estradiol concentrations of 1, 100, and 10,000 pg/mL of as indicated. Outliers shown are values below or above 1.5 × Interquartile Range from 1st or 3rd quartile, respectively. Statistical significance calculated from Kruskal–Wallis test and Dunn’s test *post hoc* with α = 0.05. ^#^*P* = 0.1–0.05, *P < 0.05, **P < 0.01. Media control *n* = 8, *P. somerae* only *n* = 5, 1 pg/mL *n* = 5, 100 pg/mL *n* = 5, 10,000 pg/mL *n* = 5. **(A)** Acetic acid, **(B)** Butyric acid, **(C)** Propanoic acid, **(D)** Isobutyric acid, **(E)** Isovaleric acid, **(F)** Valeric acid, **(G)** Isocaproic acid, **(H)** Hexanoic acid.

In addition to measuring relative changes in citric acid cycle intermediates, we measured levels of short-chain fatty acids both in the presence and absence of 17β-estradiol. Our results show that acetic, butyric, propanoic, isobutyric, and isovaleric levels were significantly increased in solution after 8.5 h of *P. somerae* growth relative to untreated controls (Figures 3A–E, *P* < 0.05). While valeric acid levels were slightly increased after *P. somerae* growth relative to untreated controls, no significant changes were observed with estradiol (Figure 3F, *P* > 0.05, α = 0.05). Additionally, no changes were observed in isocaproic acid levels after *P. somerae* growth (Figure 3G, *P* > 0.05). Apart from hexanoic acid levels being slightly decreased at 17β-estradiol concentrations of 10,000 pg/mL most likely due to experimental variation (Figure 3H, *P* > 0.05), 17β-estradiol has no statistically significant impact on short chain fatty acid production relative to untreated *P. somerae* in chopped meat broth.

### *Porphyromonas somerae* Invades Human Endometrial Cancer Cells

**FIGURE 4:**
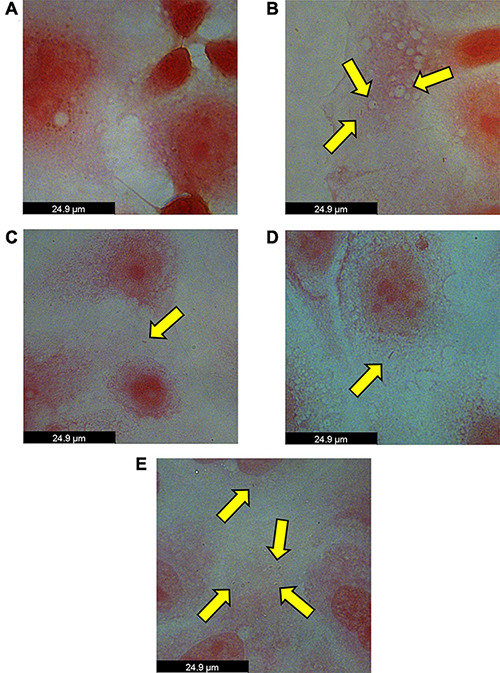
Brightfield images of intracellular *Porphyromonas somerae* noted by yellow arrows after an invasion assay with KLE cells. Invasion assay was performed at an MOI of 100:1 for 4 h at 5% O_2_ and 37°C in KLE media, followed by an antibiotic treatment for 4 h, washing, fixation, and Gram staining. Images were taken using a 100× objective under immersion oil and 10× ocular objective for a final magnification of 1,000×. **(A)** KLE cells without *P. somerae*. **(B)** KLE cells with *P. somerae*. **(C)** KLE cells with *P. somerae* and 1 pg/mL of 17β-estradiol. **(D**) KLE cells with *P. somerae* and 100 pg/mL of 17β-estradiol. **(E)** KLE cells with *P. somerae* and 10,000 pg/mL of 17β-estradiol.

To test our hypothesis that *P. somerae* invades host cells similar to *P. gingivalis*, we performed an *in vitro* invasion assay of *P. somerae* with endometrial cancer (KLE) cells at 5% O_2_, simulating levels present in endometrial tissue to study pathogenic interaction. Prior to performing the invasion assay, we confirmed that neither antibiotics nor 17β-estradiol would have an observable impact on KLE cell growth during the time of the invasion assay. After exposing KLE cells to comparable oxygen, antibiotic, and 17β-estradiol levels to be used in invasion assays, we found no statistically significant impact on growth (Supplementary Figure 2, *P* > 0.05). After a 4-h incubation to allow for infection with a multiplicity of infection (MOI) of 100:1, KLE cells with intracellular *P. somerae* were visualized by Gram staining and brightfield microscopy (visual outline of invasion assay and Gram staining protocol; Supplementary Figure 3A). Apparent Gram-negative rod-shaped infiltrates, characteristic of *P. somerae*, were observed in the cytosol of treated KLE cells relative to KLE cells only treated with antibiotics (Figures 4A,B). Additionally, zones of clearing were observed surrounding rod-shaped infiltrates, suggesting capsule formation. In preliminary experiments, additional MOIs including 10:1 and 1,000:1 were tested for optimized invasion. Using an MOI of 10:1 resulted in no notable presence of *P. somerae* after infection, while 1,000:1 resulted in rapid KLE cell death beyond measurable use (data not shown).

Considering the increased growth rates observed in *P. somerae* upon exposure to 17β-estradiol, we also hypothesized that 17β-estradiol may alter virulence and host-pathogen interaction. To study this, we performed an *in vitro* invasion assay of *P. somerae* with KLE cells using the same methods as Figures 4A,B, but with added 17β-estradiol. Potential intracellular *P. somerae* were detected at estradiol concentrations of 1, 100, and 10,000 pg/mL (Figures 4C–E).

### *Porphyromonas somerae* Is Recovered From Invasion Assays With Endometrial Cancer (KLE) Cells

**FIGURE 5:**
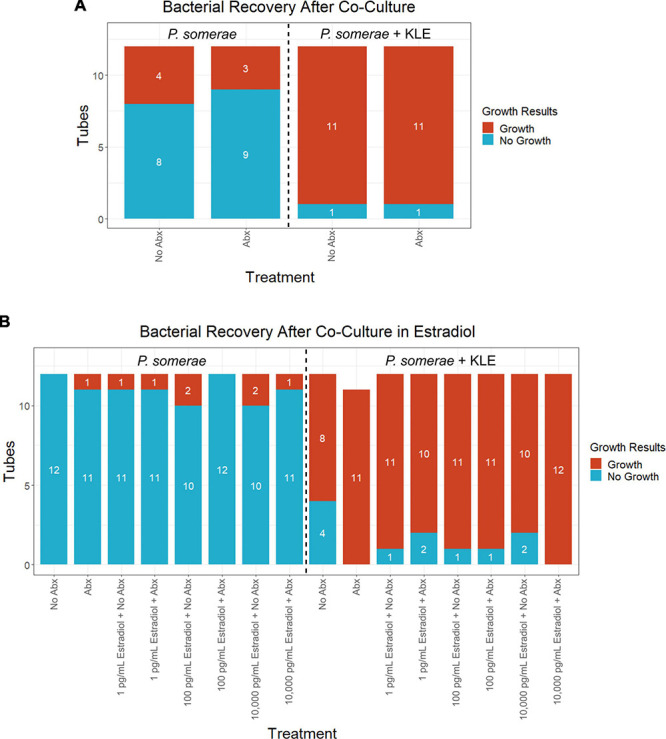
Frequency of *Porphyromonas somerae* recovered from KLE cell lysate from invasion assay after washing with or without antibiotics (Abx). Tubes monitored for bacterial growth for 14 days. All experiments performed twice with *n* = 6 for each treatment for a total *n* = 12. Statistical analysis was performed using a binary logistic regression analysis. **(A)** Invasion assay of *P. somerae* + KLE cells with and without 4-h antibiotic treatment prior to 4-h incubation. Total significance between cultures with and without KLE, *P* = 1.44 × 10^–4^. **(B)** Invasion assay with increasing 17β-estradiol concentrations. Total significance between cultures with and without KLE, *P* < 2 × 10^–16^.

After finding visual evidence for intracellular invasion, we used a quantitative approach for assessing intracellular invasion frequency. This approach also allowed us to ensure that we were observing bacterial structures as opposed to artifact or contamination. Using the same invasion assay technique as reported in the previous section, we co-incubated *P. somerae* with or without KLE cells. After washing and antibiotic treatments, KLE cells were lysed to liberate intracellular bacteria. This was followed by transfer of the lysate to pre-reduced chopped meat broth. Cultures were then monitored daily for growth for 14 days (visual outline of invasion assay; Supplementary Figure 3B). Using binary logistic regression analysis, we found that a significantly increased frequency of *P. somerae* was recovered after 14 days in wells that were co-incubated with KLE cells relative to those without KLE cells regardless of antibiotic treatment (Figure 5A). We then used the same technique to quantify the relative frequency of *P. somerae* recovered when KLE cells were co-incubated with *P. somerae* in the presence of added 17β-estradiol. We again found a significant difference using binary logistic regression analysis between treatments with and without KLE (*P* < 0.001). However, we observed no significant differences in recovered *P. somerae* in the absence versus presence of any added 17β-estradiol (Figure 5B, *P* > 0.05). During the 14-day period after the invasion assay, atypical growth phenotypes observed in chopped meat broth were checked for contamination by DNA extraction and Sanger sequencing of the hypervariable region of the 16S rRNA gene. One replicate tube from our invasion assay using KLE with *P. somerae* followed by antibiotic treatment and washing during the added 17β-estradiol experiment was found to be contaminated with *Bacillus nealsonii*.

### *Porphyromonas somerae* Shares Similar Intracellular Invasion and Persistence Mechanisms With *Porphyromonas gingivalis*

Upon examination of orthologous genes in *P. somerae* using OrthoFinder, we identified that it contains the same components for fumarate and succinate driven energy production as *P. gingivalis* (Supplementary Table 1). We also observed conserved endopeptidase genetic components between *P. somerae* and *P. gingivalis*, indicating that essential post-invasion cell survival pathways are conserved (Supplementary Table 2). We were not able to identify internalin-like proteins or gingipain-like proteins within the ortholog groups. However, we have previously identified an alternative proteolytic virulence factor, streptopain, in the transcriptome of *P. somerae* (Liu et al., 2019). Additionally, we have previously found fumarate reductase and NADP^+^-dependent malic enzyme mRNA transcripts *in vitro* (Liu et al., 2019) to facilitate succinate and malate production, respectively. We also identified orthologous genes in *P. somerae* that indicate ability for capsule formation in the form of the CapM gene, a capsular polysaccharide synthesis protein (Supplementary Table 3). We have also previously identified capsule formation precursor mRNA transcripts *in vitro* for the polysaccharide export protein Wza (Liu et al., 2019). Components such as Wza which are involved in capsule formation play important roles in virulence (Niu et al., 2020). The presence of mechanisms for intracellular capsular polysaccharide production and further export indicate capability of *P. somerae* to form a capsule.

## Discussion

Capsule formation and *P. somerae* internalization was suggested by the observed safranin retention in the cytosol of KLE cells and zone of clearing observed around the bacillus shaped structure within the KLE cell cytosol. Coupled with the increased frequency of bacterial recovery from KLE cell lysate after co-culturing with *P. somerae* relative to wells without KLE cells, we show evidence of facultative intracellular presence of *P. somerae*. Considering the shared genetic components for virulence we found between *P. somerae* and *P. gingivalis*, it is plausible that *P. somerae* is using invasive techniques comparable to that of *P. gingivalis*. While such interactions are evident as hypothesized, we were surprised to find no changes in apparent invasion frequency during exposure to various physiological 17β-estradiol levels. Considering the increased growth rate observed in the presence of 17β-estradiol when grown in pre-reduced chopped meat broth, we hypothesized that 17β-estradiol might induce a change in virulent activity. However, apart from changes in growth rate in liquid media which may indirectly contribute to virulence, we were unable to characterize any direct and immediate changes to virulence in response to 17β-estradiol.

As *P. somerae* growth had not previously been characterized in broth, we found reduced chopped meat broth to be favorable for experimental use. When grown on blood agar, differential growth patterns are observed. These differences result in patchy “satellite” colonies (Summanen et al., 2005) with vastly different growth rates. In addition to a faster growth rate achieved in reduced chopped meat broth, known CFUs relative to cell density in liquid suspension could be established for an accurate MOI in our invasion assays. For these reasons, pre-reduced chopped meat broth was used for overnight culturing of *P. somerae* in invasion assays presented here. While previous antibiotics have been shown to inhibit the growth of *P. somerae*, the MIC of gentamicin has not been shown until now.

Previous sequencing of *P. somerae* cDNA has shown production of mRNA transcripts for metabolic components of interest such as fumarate reductase subunits (Liu et al., 2019). While we did not observe a significant reduction of fumarate levels in chopped meat broth after 8.5 h of growth, we did observe increased succinate levels. This is likely the result of fumarate reductase activity. We also observed increased levels of malate which is consistent with the previous observation that NADP^+^-dependent malic enzyme is found in the transcriptome of *P. somerae*. Various short chain fatty acids were also found to be significantly enriched in the media after 8.5 h of growth. Interestingly, we did not observe significant enrichment or depletion of any metabolite when *P. somerae* was grown in 17β-estradiol relative to untreated *P. somerae*. Although we hypothesized that significant differences would be observed due to increased growth rate, and therefore metabolic activity, no 17β-estradiol treatments yielded significant differences.

In summary, our results show that *P. somerae*, a microorganism we have previously associated with endometrial cancer (Walther-António et al., 2016; Walsh et al., 2019) is capable of invading endometrial cells in both the presence and absence of estradiol. This suggests that the presence of the bacteria in the endometrial lining presents the possibility of virulent action. While intracellular invasion in response to extracellular stressors such as antimicrobials is an established phenomenon in bacteria (Mu et al., 2016), we found no significant differences in bacterial recovery post-invasion assay with or without antibiotic exposure. This suggests invasion is uncoupled from antibiotic exposure in our *in vitro* experimental model. In addition to the capability of invasion, *P. somerae* also demonstrates the ability to produce metabolites that can interfere with the normal functioning of host cells, namely succinate. Succinate production by *P. gingivalis* has been shown to interfere with the stability of the hypoxia inducible factor HIF–1α, triggering host inflammatory responses (Su et al., 2020). Importantly, intracellular succinate excess is well known to stabilize the HIF–1α complex by inhibiting prolyl hydroxylase domain enzymes (PHDs) (King et al., 2006). Because HIF–1α stability and its upregulation is a hallmark of multiple cancers, including endometrial cancer (Chen et al., 2020), it is reasonable to consider that *P. somerae* may be a contributor to this phenomenon by invading cells and generating an excess of succinate in the host cell akin to the effects of a mitochondrial mutation in succinate dehydrogenase or fumarate reductase genes. In addition to the possibility of *P. somerae* succinate production interfering with HIF-1α stability, other factors related to EC development may be considered for future study. Specifically, intracellular persistence may differentially affect host cell epigenetic processes such as methylation patterns. Hypermethylation of key loci associated with development of EC has been previously reported and may be influenced by host-associated bacteria (Multinu et al., 2020).

In light of these possibilities, it is clearly important to dedicate future research efforts toward the determination of the persistence of these cellular invasions and define their impact on host cells. Distinguishing the impact of the invasions in transformed and benign cells will also provide further insight into host-bacteria interactions and their meaning in the context of the development and/or progression of endometrial cancer. It will also be of relevance to further scrutinize factors that may be conducive or favorable to *P. somerae*’s invasive behavior, such as gradients of iron, which are known to stimulate bacterial virulence. It will be equally relevant to discern the role of other microbiome components that accompany this microorganism and add this new dimension of knowledge to our collective understanding of endometrial cancer and opportunities for intervention.

## Materials and Methods

### Cell Maintenance

#### KLE Growth and Maintenance

ATCC^®^ CRL-1622^TM^ KLE human endometrial adenocarcinoma cells were used in all experiments pertaining to endometrial cells. Gibco^TM^ DMEM/F-12 GlutaMAX^TM^ with phenol red and added 10% fetal bovine serum (FBS) before filter sterilization was used to maintain KLE cell cultures. All KLE cells maintained at 37°C in 5% CO2. KLE cells were washed with PBS and fresh medium was added every 48–72 h. Cells were passaged at 85–100% confluency. Prior to splitting, old culture media was aspirated off, then washed with pre-warmed 1× PBS. PBS was then aspirated off and 4 mL Gibco^TM^ 0.25% Trypsin-EDTA (1×) added to culture flask, followed by a 7–12 min incubation at 37°C. Upon completion of incubation, 11 mL of fresh pre-warmed media was added to neutralize trypsin activity yielding a final volume of 15 mL freely suspended cells in media for plating.

#### *Porphyromonas somerae* Growth and Maintenance

*Porphyromonas somerae* (ATCC^®^ BAA-1230^TM^) was grown in BD BBL^TM^ Pre-Reduced Chopped Meat Carbohydrate Broth (Becton, Dickinson, and Company, Franklin Lakes, NJ) for maintenance of cells, growth curves, minimum inhibitory antibiotic concentrations, impact of 17β-estradiol on bacterial growth and metabolomics, and recovery of viable bacteria post-invasion assay. Stocks used to grow overnight cultures were preserved at −80°C in 25% sterile glycerol.

### Growth Curves and Minimum Inhibitory Concentrations

#### Minimum Inhibitory Concentration of Gentamicin Growth Curve

Initial culture of *P. somerae* was grown overnight in chopped meat broth in anaerobic conditions at 37°C. Ten microliter inoculums of overnight culture with an OD_600_ measurement of 0.718 standardized to blank chopped meat broth were transferred to fresh chopped meat broth in final volumes of 4 mL. Growth was assessed in gentamicin concentrations of 1, 10, 100 μg/mL, or no gentamicin in addition to tubes containing media only controls. All experimental conditions were tested using 3 replicates of each. Growth was assessed at 37°C in anaerobic conditions. Every 2 h, tubes were inverted to resuspend cultures, and small aliquots were transferred to clean UVette^®^ cuvettes (Eppendorf, Hamburg, Germany). OD_600_ measurements were taken using a BioPhotometer (Eppendorf, Hamburg, Germany) every 2 h for 24 h to assess cell density.

#### Growth Curve to Determine Viable CFUs/mL

Initial culture of *P. somerae* was grown overnight in chopped meat broth in anaerobic conditions at 37°C. Anaerobic conditions were maintained at <30 ppm of O_2_ and 2.5–3% H_2_ (COY Lab Products Inc., Grass Lake, MI). Ten μL of overnight culture were used to inoculate chopped meat broth tubes in triplicate. All samples were homogenized by inverting tubes prior to sub-sampling for OD_600_ analysis. All samples were analyzed at *t* = 0 and every 4 h for 16 h, then a final reading at 24 h post inoculation. At intervals of analysis, 10-fold serial dilutions were performed sequentially 6 times for a final global dilution factor of 1 × 10^–6^. At each interval, OD_600_ measurements were taken from undiluted samples and all serial dilutions using a BioPhotometer (Eppendorf, Hamburg, Germany). Additionally, 50 μL of undiluted samples and all serial dilutions were transferred to BD-BBL CDC Anaerobe 5% SB Blood Agar Plate (Becton, Dickinson, and Company, Franklin Lakes, NJ) and incubated in anaerobic conditions at 37°C to allow for the formation of colony forming units. After 5 days, plates from dilutions yielding distinguishable colony forming units were counted. CFUs/mL at each timepoint were determined from plates with discernable colonies that could be quantified.

### Targeted Metabolomics

#### Sample Preparation

Initial culture of *P. somerae* was grown overnight in chopped meat broth in anaerobic conditions at 37°C. Chopped meat broth with added 17β-estradiol was prepared using cyclodextrin-encapsulated 17β-estradiol (Sigma-Aldrich, St. Louis, MO). Cyclodextrin-encapsulated 17β-estradiol was used to allow for increased solubility in water-based solutions. Stocks were prepared by weighing out 15 mg of dry reagent yielding approximately 0.7125 mg 17β-estradiol per manufacturer’s description. Dry reagent was subsequently dissolved in chopped meat broth. Overnight culture of *P. somerae* was removed from the incubator and OD_600_ measurements taken to assess initial concentration. Culture was further diluted in pre-reduced chopped meat broth to achieve OD_600_ measurements of 1.39. In anaerobic conditions, 50 μL of diluted overnight culture was transferred to 950 μL of chopped meat broth with 17β-estradiol in sterile microcentrifuge tubes to achieve a final volume of 1 mL. At final volumes, 17β-estradiol concentrations of either 1, 100, 10,000 pg/mL, or no 17β-estradiol were achieved. Treatments were incubated at 37°C in anaerobic conditions for 8.5 h. Additional aliquots of no added *P. somerae* controls were incubated with experimental treatments for metabolomic analysis of media. Treatments were then removed from the incubator and centrifuged (5,000 × g, 5 min) to separate media from bacterial cells. Carefully avoiding the pellet, supernatant was removed and transferred to sterile microcentrifuge tubes. Samples were preserved at −80°C until targeted metabolomics was performed.

#### Short-Chain Fatty Acid Assessment via Gas Chromatography-Mass Spectrometry

Short-chain fatty acids were quantitated via gas chromatography-mass spectrometry (GC-MS) as previously published (Hale et al., 2018a, b) with a few modifications. Briefly, 50 μL media was added to a tube containing 10 μL internal standard solution at pH 2 containing 2-ethylbutyric acid, d5-propionic acid d7-butyric acid, d9-valeric acid, and d11-caproic acid. 300 μL of dichloromethane (DCM) was used to extract short-chain fatty acids from the mixture. The extract was derivatized with N-Methyl-N-tert-butyldimethylsilyltrifluoroacetamide (MTBSTFA) prior to analysis on a gas chromatograph-mass spectrometer. Derivatized analytes were separated on a DB5MS column (30 m × 0.25 mm ID × 0.25 μm film thickness) prior to entering the mass spectrometer detector (Agilent MSD5977A). Concentrations of acetic acid (m/z 117.0), propionic acid (m/z 131.1), isobutyric acid (m/z 145.1), butyric acid (m/z 145.1), isovaleric acid (m/z 159.1), valeric acid (m/z 159.1), isocaproic acid (m/z 173.2), and hexanoic acid (m/z 173.2) were measured against 12-point calibration curves that underwent the same derivatization.

#### Citric Acid Cycle Assessment via Gas Chromatography-Mass Spectrometry

Concentration of citric acid cycle analytes were measured by GC-MS as previously described (Gonsalves et al., 2018; Wilkins et al., 2019) with a few modifications. Briefly, 50 μL media was extracted in 300 μL of chilled methanol and acetonitrile solution after the addition of 20 μL of internal solution containing U-^13^C labeled analytes. After drying the supernatant in the speed vacuum, the sample was derivatized with ethoxime and then with MtBSTFA + 1% tBDMCS (N-Methyl-N-(t-Butyldimethylsilyl)-Trifluoroacetamide + 1% t-Butyldimethylchlorosilane) before it was analyzed on an Agilent 5977B gas chromatograph-mass spectrometer under electron impact and single ion monitoring conditions. Concentrations of lactic acid (m/z 261.2), fumaric acid (m/z 287.1), succinic acid (m/z 289.1), ketoglutaric acid (m/z 360.2), malic acid (m/z 419.3), aspartic acid (m/z 418.2), 2-hydroxyglutaratic acid (m/z 433.2), cis aconitic acid (m/z 459.3), citric acid (m/z 591.4), isocitric acid (m/z 591.4), and glutamic acid (m/z 432.4) were measured against a 7-point calibration curve that underwent the same derivatization.

### Impact of 17β-Estradiol on Growth

#### Impact of 17β-Estradiol on *P. somerae* Growth

Initial culture of *P. somerae* was grown overnight in chopped meat broth in anaerobic conditions at 37°C. Chopped meat broth with added 17β-estradiol was prepared using the same method as described in targeted metabolomics sample preparation. Overnight culture of *P. somerae* was removed from the incubator and an OD_600_ measurement was taken to assess initial concentration using a BioPhotometer (Eppendorf, Hamburg, Germany). Cultures were diluted to achieve OD_600_ measurement of 1.39. In anaerobic conditions, 50 μL of diluted overnight culture was transferred to 950 μL of chopped meat broth with 17β-estradiol in sterile microcentrifuge tubes to achieve a final volume of 1 mL. At final volumes, 17β-estradiol concentrations of either 1, 100, 10,000 pg/mL, or no 17β-estradiol were achieved. Treatments were incubated at 37°C in anaerobic conditions for 8.5 h. Cultures were then removed from anaerobic conditions and vortexed briefly. Cell density was then assessed via OD_600_ measurements.

#### Impact of 17β-Estradiol and Antibiotics on KLE Cell Growth

KLE cells were grown in 6-well plates to approximately 90–95% confluency. Prior to treatment, old media was aspirated off and wells washed with PBS. Wells were then treated with filter sterilized Gibco^TM^ DMEM/F-12 GlutaMAX^TM^ (without phenol red) supplemented with 10% FBS and 17β-estradiol concentrations of either 1, 100, 10,000 pg/mL, or no 17β-estradiol. In addition, all treatments of 17β-estradiol were tested in the combined presence or absence of gentamicin (200 μg/mL) and cefoxitin (200 μg/mL). All plates were then placed into hypoxic gas chambers (10% CO_2_, 5% O_2_, 85% N_2_; gas mixture Praxair, Danbury, CT) at 37°C for 8.5 h. After incubation, cells were removed from hypoxic conditions and washed with PBS. Wells were then treated with Gibco^TM^ 0.25% Trypsin-EDTA (1x) for 9–13 min at 37°C until KLE cells were freely suspended in solution. Trypsin-EDTA was subsequently neutralized with 2× volume Gibco^TM^ DMEM/F-12 GlutaMAX^TM^ (without phenol red) supplemented with 10% FBS, and gently pipetted to break up cell aggregates. Cell density was then assessed via OD_600_ measurements using a BioPhotometer (Eppendorf, Hamburg, Germany).

### Invasion Assay of *P. somerae* and Endometrial Cancer (KLE) Cells

#### Inoculum Preparation

Prior to the assay, *P. somerae* was grown overnight in chopped meat broth to reach mid-log phase for maximized invasion frequency comparable to *P. gingivalis* (Lamont et al., 1995). To prepare the inoculum, *P. somerae* was transferred to microcentrifuge tubes and washed 3× via centrifugation (5,000 × g, 90 s) followed by resuspension in PBS. The final resuspension was in filter sterilized Gibco^TM^ DMEM/F-12 GlutaMAX^TM^ (without phenol red) supplemented with 10% FBS, and the contents were transferred to a sterile 50 mL conical tube. This suspension was then diluted to an approximate MOI of 100:1 based on OD_600_ measurements using a BioPhotometer (Eppendorf, Hamburg, Germany). MOI of 100:1 was used to maximize invasion frequency as seen in *P. gingivalis* (Lamont et al., 1995). Phenol red was excluded from the media to prevent artificial estrogen receptor agonistic activity (Berthois et al., 1986). When indicated, an inoculum of *P. somerae* was resuspended in media containing 17β-estradiol (Sigma-Aldrich, St. Louis, MO) similar to methods used in the impact of 17β-estradiol on bacterial growth. Prior to experiments that involved Gram staining, KLE cells were grown on sterile coverslips. Coverslips were pre-treated for 2 h with 0.1% (W/V) poly-L-lysine (Sigma-Aldrich, St. Louis, MO), followed by washing in sterile PBS. All invasion assays used KLE cells that were grown to approximately 90–95% confluency.

#### Infection

Media was aspirated from KLE cells followed by one wash with PBS. The *P. somerae* inoculum was then added to each well as appropriate. When making the no-*P. somerae* control wells, filter sterilized Gibco^TM^ DMEM/F-12 GlutaMAX^TM^ (without phenol red) supplemented with 10% FBS was added. All plates were then put in hypoxic gas chambers with a 10% CO_2_, 5% O_2_, 85% N_2_ gas mixture (Praxair, Danbury, CT) at 37°C for 4 h. After 4 h of infection under hypoxic conditions to maximize invasion frequency but restrict bacterial growth (Lamont et al., 1995), the wells were washed 3× with PBS, followed by addition of Gibco^TM^ DMEM/F-12 GlutaMAX^TM^ (without phenol red) supplemented with 10% FBS, cefoxitin (200 μg/mL), and gentamicin (200 μg/mL). The antibiotic treatment was conducted for 4 h at 37°C in hypoxic conditions as used in the previous incubation. The KLE cells were subsequently washed 3× with PBS.

#### Bacterial Recovery Post-invasion

After washing with PBS, 0.1% (W/V) saponin (Sigma-Aldrich, St. Louis, MO) was added (700 μL/well) for 20 min at room temperature to permeabilize KLE cells disrupting cell membrane integrity (Amidzadeh et al., 2014). Saponin treated well contents were mixed thoroughly via repeated suspension and mechanical disruption with a sterile needle and syringe to liberate KLE cells adherent to the coverslip and release cell contents. Total well contents were transferred to chopped meat broth tubes via sterile needle and syringe, then permitted to incubate in anaerobic conditions at 37°C for 14 days to monitor for bacterial growth. At the end of 14 days, tubes were examined visually for turbidity. Chopped meat broth tubes with apparent growth within 14 days were considered to be evident of *P. somerae* recovery after invasion.

#### Assessment by Gram Stain

After washing, wells were fixed with 4% paraformaldehyde in PBS (Santa Cruz Biotechnology, Dallas, TX) for 1.5 h at room temperature. After fixation, paraformaldehyde was aspirated off and washed twice with PBS. PBS was then removed in preparation for Gram staining. Fixated coverslips were removed from 12-well plates and transferred to clean paper towels. Gram stain performed using the HARLECO^®^ Complete Bacterial Staining Kit (Sigma-Aldrich, St. Louis, MO) by adding crystal violet to coverslips for 60 s, followed by washing with deionized (DI) water. Coverslips were then treated with iodine for 60 s, followed by washing with DI water. Coverslips were decolorized for 20 s, followed by a DI water wash. A final treatment of safranin counterstain was applied for 60 s followed by a wash with DI water. Coverslips were transferred to microscope slides under immersion oil. Brightfield microscopy imaging was then performed on an automated Leica DMi8 advanced microscope with LAS X software controls (Leica, Wetzlar, Germany). Images were taken using a 100× objective and 10× ocular objective for a final magnification of 1,000×.

### Computational Analysis of Genomic Components of *Porphyromonas somerae*

Orthologous genes across two strains of *Porphyromonas gingivalis, Porphyromonas levii*, and *Porphyromonas somerae* were compared using OrthoFinder (Emms and Kelly, 2015). The default thresholds specified in the workflow manual were used. A previously published annotated assembly for *P. somerae* was used during orthogroup comparison and can be found under the NCBI RefSeq accession NZ_AQVC01000001.1. Previously published RNA sequencing data was used to identify the genes of interest expressed *in vitro.* Original annotations were generated in Prokka and are represented by analogous RefSeq IDs (Seemann, 2014; Brettin et al., 2015; Liu et al., 2019).

### Data Analyses

#### Statistical Analyses

Statistical analysis was performed in R version 4.0.2 and Rstudio version 1.3.1073. Kruskal–Wallis tests were performed due to assumptions of normality not being met. The Kruskal–Wallis test was performed using the kruskal.test function in R. Dunn’s tests were performed using the dunn.test R package version 1.3.5 (Dinno, 2017). Growth curves of *P. somerae* in gentamicin were analyzed using the growthcurver R package version 0.3.0 (Sprouffske, 2018), dplyr R package version 1.0.1 (Wickham et al., 2020), reshape2 R package version 1.4.4 (Wickham, 2020). Binary logistic regression analyses were completed using R packages MASS version 7.3-54 and pROC version 1.17.0.1 (Venables and Ripley, 2002; Robin et al., 2011). An α = 0.05 was used for all statistical analyses. Any *p* ≤ 0.05 was considered significant. Any statistical analysis with an adjusted *p*-value was used to determine significance. Full statistical analyses available (Supplementary Table 4).

#### Figure Production

Graphs were generated using R version 4.0.2 and Rstudio version 1.3.1073. Growth curves and bacterial recovery after co-culturing was graphed using the ggplot2 R package version 3.3.3 (Wickham, 2016). Axis labels were modified using Inkscape version 1.0. The bacterial growth in 17β-estradiol graph, KLE cell growth in 17β-estradiol and antibiotics graph, and metabolomics graphs were generated using the boxplot function in R. Axis labels, statistics, and graph breakages were added using Inkscape version 1.0. Yellow arrows in Gram stained brightfield microscopy images were added using Inkscape version 1.0. Images were cropped with the scale bar maintained at equal size to center images around areas of interest using Inkscape version 1.0.

## Data Availability Statement

The datasets presented in this study can be found in online repositories. The names of the repository/repositories and accession number(s) can be found below: Figshare. Link - https://figshare.com/projects/Porphyromonas_somerae_Invasion_of_Endometrial_Cancer_Cells/97196.

## Author Contributions

TC, JM, DW, NC, WC, SK, and MW-A conceived and designed the experiments. TC, JM, and DW performed the experiments. TC, JM, DW, WH, and PJ performed the data analyses. WH and PJ assisted in finding genomic and transcriptomic components of interest. TC, JM, DW, WH, and MW-A contributed to writing the manuscript. All authors contributed to the article and approved the submitted version.

## Conflict of Interest

MW-A is a member of the scientific advisory board of LUCA Biologics, Inc. on research related to urinary tract infections, preterm birth, and reproductive medicine. These activities do not overlap with the research presented here. All remaining authors declare that the research was conducted in the absence of any commercial or financial relationships that could be construed as a potential conflict of interest.

## Publisher’s Note

All claims expressed in this article are solely those of the authors and do not necessarily represent those of their affiliated organizations, or those of the publisher, the editors and the reviewers. Any product that may be evaluated in this article, or claim that may be made by its manufacturer, is not guaranteed or endorsed by the publisher.
