# Supplementary material for: Porphyromonas somerae Invasion of Endometrial Cancer Cells
Source: Front Microbiol. 2021 Jul 23;12:674835. doi: 10.3389/fmicb.2021.674835 (PMC8343132; doi:10.3389/fmicb.2021.674835)
Supplement: Supplementary file 1 [file Data_Sheet_1.zip › Data Sheet 1.PDF]

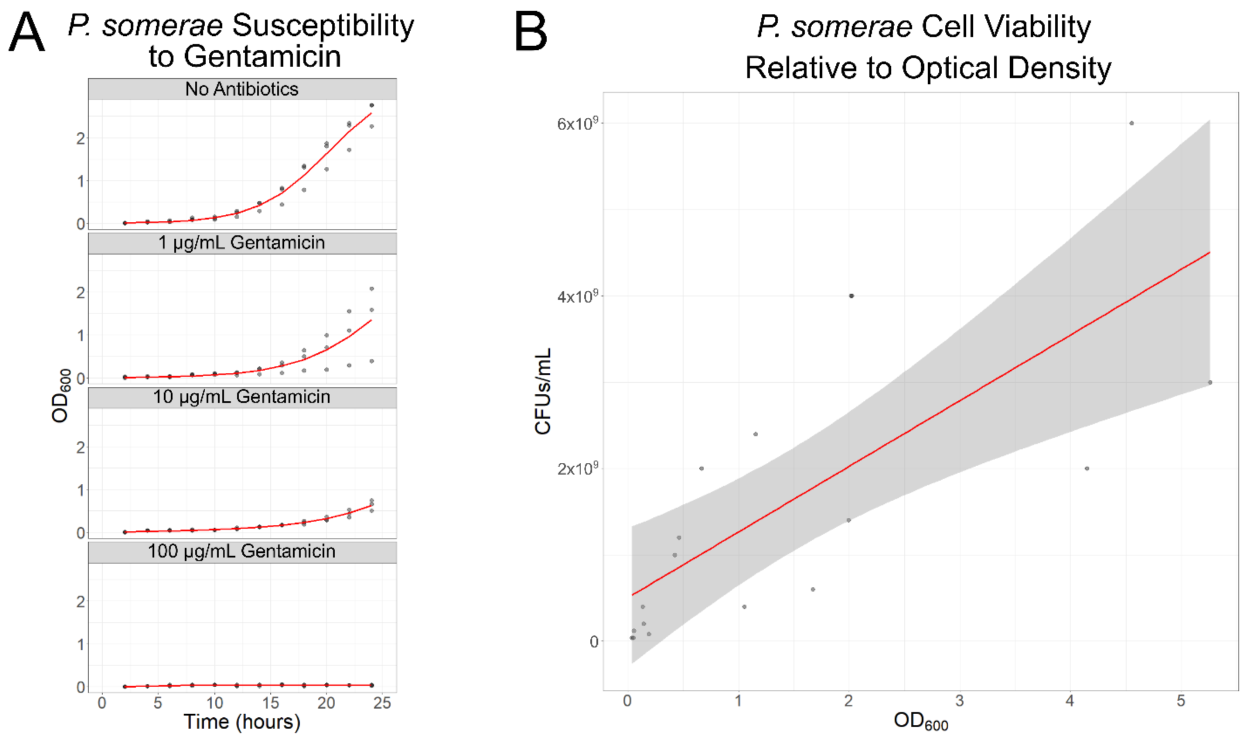

Supplementary Figure 1 – (A) OD_{600} measurements in chopped meat broth over 24-hour growth curve of *Porphyromonas somerae* in no antibiotics or gentamicin concentrations of 1 µg/mL, 10 µg/mL, 100 µg/mL. n=3 for each treatment. **(B)** Calculated CFUs/mL of *P. somerae* from counted colonies plated onto blood agar during separate 24-hour growth curves with n=3 performed in parallel. Gray zone indicates 95% confidence interval for best fit line.
