# Supplementary material for: Porphyromonas somerae Invasion of Endometrial Cancer Cells
Source: Front Microbiol. 2021 Jul 23;12:674835. doi: 10.3389/fmicb.2021.674835 (PMC8343132; doi:10.3389/fmicb.2021.674835)
Supplement: Supplementary file 1 [file Data_Sheet_1.zip › Data Sheet 2.PDF]

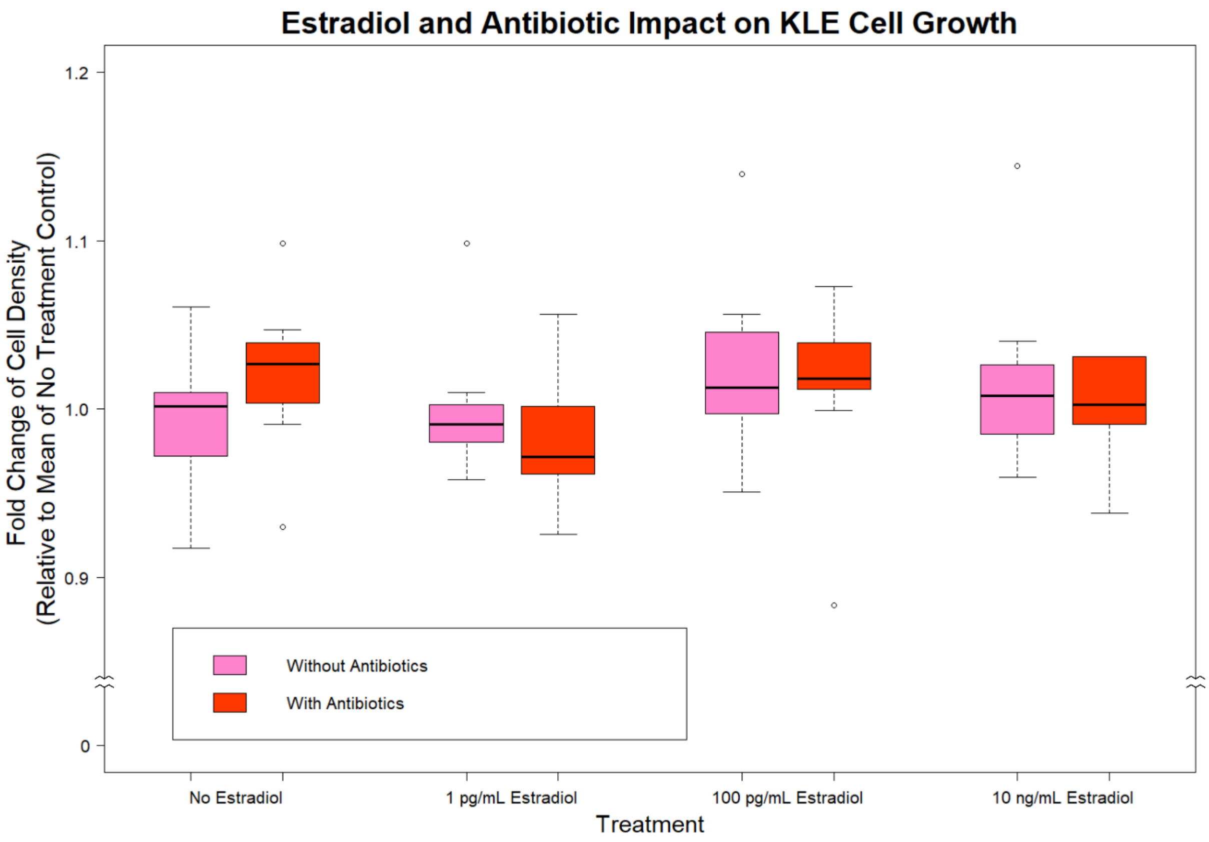

Supplementary Figure 2 – Fold change in KLE cell density after 8.5-hour exposure to 17β-estradiol or (200 µg/mL) of gentamicin and (200 µg/mL) of cefoxitin as indicated. Fold change determined by comparing OD_{600} measurement to the corresponding experimental mean OD_{600} of no 17β-estradiol or antibiotic control. Experiment performed 3 times with n=3 for each treatment for a total n=9 for all samples. No significance observed from Kruskal–Wallis test with $\alpha = 0.05$.
