# Supplementary material for: Porphyromonas somerae Invasion of Endometrial Cancer Cells
Source: Front Microbiol. 2021 Jul 23;12:674835. doi: 10.3389/fmicb.2021.674835 (PMC8343132; doi:10.3389/fmicb.2021.674835)
Supplement: Supplementary file 1 [file Data_Sheet_1.zip › Data Sheet 3.PDF]

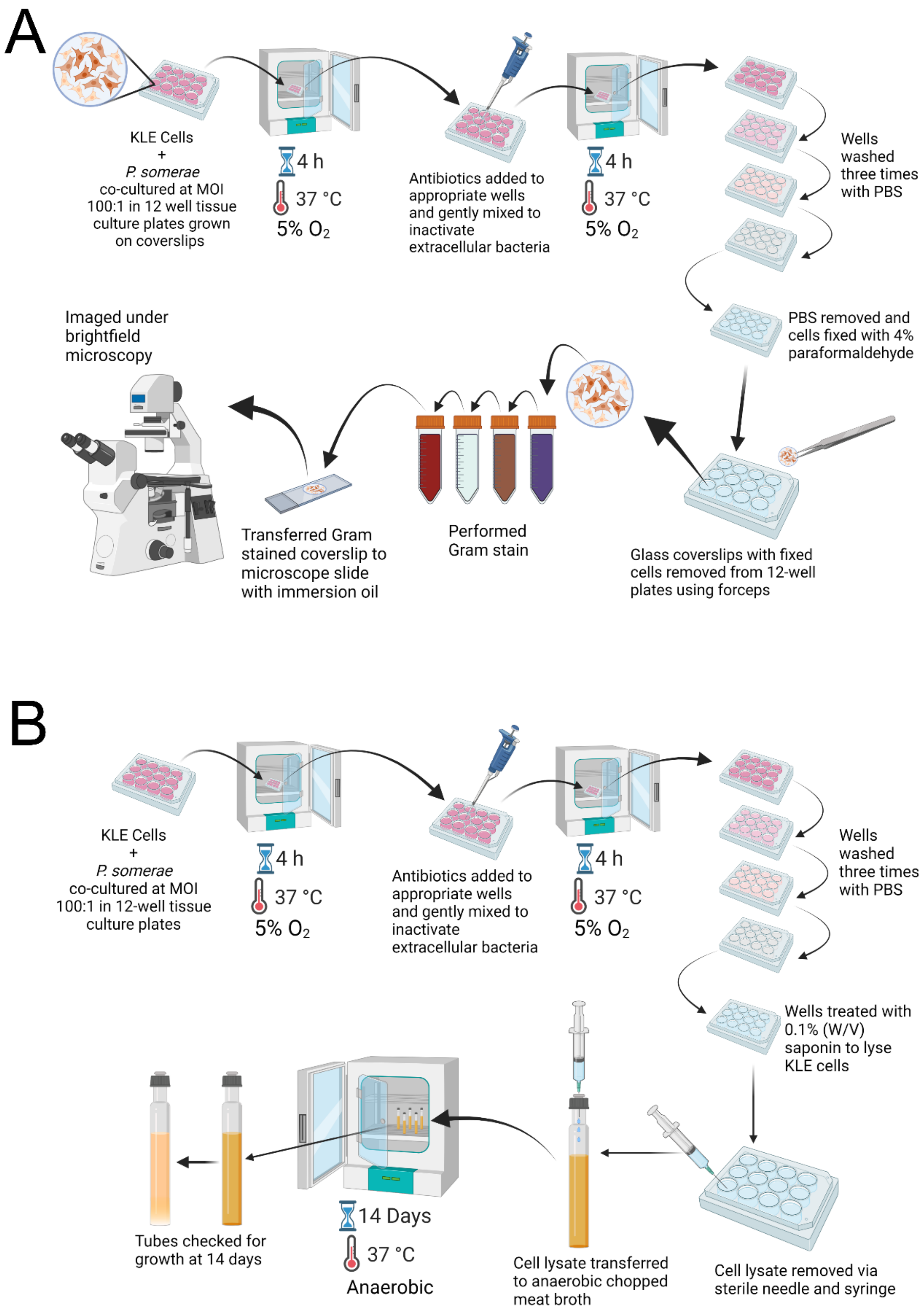

Supplementary Figure 3 – Visual outline of invasion assay protocol. Created with BioRender.com **(A)** Invasion assay and Gram staining to visualize intracellular bacteria via brightfield microscopy. **(B)** Invasion assay with cell lysis to measure frequency of *P. somerae* recovery.
